# Anti-inflammatory Mechanisms Triggered by Apoptotic Cells during Their Clearance

**DOI:** 10.3389/fimmu.2017.00909

**Published:** 2017-08-02

**Authors:** Zsuzsa Szondy, Zsolt Sarang, Beáta Kiss, Éva Garabuczi, Krisztina Köröskényi

**Affiliations:** ^1^Department of Biochemistry and Molecular Biology of Medical Faculty, University of Debrecen, Debrecen, Hungary; ^2^Department of Basic Medical Sciences of Dental Faculty, University of Debrecen, Debrecen, Hungary

**Keywords:** apoptotic cell, phagocytosis, anti-inflammatory, pro-resolving lipid mediators, phosphatidylserine

## Abstract

In the human body, billions of cells die by apoptosis every day. The subsequent clearance of apoptotic cells by phagocytosis is normally efficient enough to prevent secondary necrosis and the consequent release of cell contents that would induce inflammation and trigger autoimmunity. In addition, apoptotic cells generally induce an anti-inflammatory response, thus removal of apoptotic cells is usually immunologically silent. Since the first discovery that uptake of apoptotic cells leads to transforming growth factor (TGF)-β and interleukin (IL)-10 release by engulfing macrophages, numerous anti-inflammatory mechanisms triggered by apoptotic cells have been discovered, including release of anti-inflammatory molecules from the apoptotic cells, triggering immediate anti-inflammatory signaling pathways by apoptotic cell surface molecules *via* phagocyte receptors, activating phagocyte nuclear receptors following uptake and inducing the production of anti-inflammatory soluble mediators by phagocytes that may act *via* paracrine or autocrine mechanisms to amplify and preserve the anti-inflammatory state. Here, we summarize our present knowledge about how these anti-inflammatory mechanisms operate during the clearance of apoptotic cells.

## Introduction

Timed initiation of apoptotic type of cell death followed by prompt removal carried out by professional engulfers or by non-professional neighboring cells plays a central role in the maintenance of tissue homeostasis. Every day billions of our cells die and get removed without inducing inflammation and autoimmunity (1). And even when inflammation is induced, efficient clearance of apoptotic neutrophils by engulfing macrophages during the inflammatory response is a determining event in initiating resolution of inflammation and contributes to the tissue repair processes following injury (2–5).

To initiate effective clearance, apoptotic cells attract engulfing cells by secreting various chemotactic signals, such as CX3CL1/fractalkine (6), lysophosphatidylcholine (7), sphingosine-1-phosphate (8), thrombospondin-1 (TSP-1) (9), ATP and UTP (10), endothelial monocyte-activating polypeptide II (11), monocyte chemoattractant protein-1 (12), cleaved human tyrosyl-tRNA synthetase (13), or the S19 ribosomal protein cross-linked dimer (14). Upon approaching the dying cells, engulfing cells must make a distinction between dead and living cells, and they act so by recognizing apoptotic cell-associated molecular patterns (ACAMPs) displayed on the cell surface of apoptotic cells (15). Most of the ACAMPs are phagocytosis initiating “eat me” signals, which are able to trigger receptors on phagocytes either directly or *via* bridging molecules. Phosphatidylserine (PS) on the outer leaflet of the cell membrane is the most universally seen “eat me” signal appearing in apoptotic cells (16). Stabilin-2, the macrophage receptor T-cell immunoglobulin- and mucin-domain-containing molecule (Tim4), and brain-specific angiogenesis inhibitor 1 have the ability to directly engage PS on dying cells (17–19), other receptors, such as Mer tyrosine kinase (MerTk) (20), scavenger receptor SCARF1 (21), or integrin αv/β3/β5 either with the CD36 (22) or with the transglutaminase 2 (TG2) coreceptors (23) require bridging molecules for PS binding. While for MerTk Gas6 and Protein S serve as bridging molecules (24, 25), TSP-1 and milk-fat globulin-E8 play a similar role for the integrin αv/β3/CD36 and integrin αv/β3/TG2 receptor complexes, respectively (22, 26, 27). In the case of apoptotic neutrophils, however, the matricellular protein CCN1 bridges PS to the αv/β3 and αv/β5 integrins of macrophages (28).

Besides the PS-recognizing bridging molecules, additional bridging molecules also contribute to the phagocytosis of apoptotic cells. Thus, serum protein C1q links Annexin A2 and A5 on the apoptotic cells (29) to both the SCARF1 scavenger receptor and to the calreticulin-associated CD91 receptor on macrophages (20, 30, 31). Apoptotic neutrophils, T cells, and human mesangial cells release annexin I (32), and annexin I enhances phagocytosis of apoptotic cells *via* a mechanism that requires FPR2/ALX receptor and its internalization (33).

Interestingly, simultaneous triggering of all these phagocytic receptors seems to result in the activation of only two evolutionary conserved signaling pathways both leading to the activation of Rac1, a small GTPase that regulates cytoskeletal rearrangements required for the phagocytosis process (34).

Uptake of apoptotic cells delivers excess materials to the phagocytes, which is degraded after the newly formed phagosome fuses with the lysosomes (35, 36). However, for full protein degradation besides the lysosomal cathepsins (35), the proteosomal pathway also has to be activated (37). In addition, to successfully metabolize lipids originated from the apoptotic cells, phagocytes also require the lipid metabolism organizing function of their lipid sensing nuclear receptors, the liver X receptors (LXRs), and the perixosome proliferator-activated receptors (PPARs) (38). LXRs and PPARs are ligand-regulated transcription factors belonging to the nuclear receptor family. They function in the form of heterodimers with the retinoid X receptors (RXRs) (39). The unligated LXRs and PPARs are located on their respective DNA response elements and recruit co-repressor molecules that repress the transcription of their target genes. Binding of their ligand, however, induces such a conformational change in their structure that results in the exchange of co-repressors for co-activators, and in the consequent start of transcription. In addition, ligated LXR and PPAR heterodimers are also capable of transrepressing genes, the transcription of which would otherwise be initiated by other transcription factors (39).

The ligands of LXRs are sterol metabolites (40, 41), while for PPARs are unsaturated fatty acids, eicosanoids, and derivatives of linoleic acid (42). Metabolically, the main function of LXRs is to regulate whole body sterol metabolism (38). Since, in mammalians, sterols cannot be degraded; in macrophages, following the degradation of apoptotic cells, activated LXRs induce the expression of cholesterol efflux transporters ABCG1 and ABCA1, Apo lipoproteins ApoC and ApoE, and the lipoprotein remodeling enzyme PLTP (43) promoting this way the efflux of apoptotic cell-derived sterols onto serum apolipoproteins and their transport to the liver, from where sterols will be redistributed again. PPARs, on the other hand, are involved in nearly every facet of fatty acid metabolism (44). Thus, PPARγ controls the expression of genes involved in lipid metabolism (43), while PPARδ controls the macrophage energy homeostasis by regulating the expression of genes involved β-oxidation, mitochondrial respiration, and thermogenesis (45).

While engulfment of a number of pathogenic targets induces a pro-inflammatory program in macrophages, uptake of apoptotic cells initiates their transition into an anti-inflammatory phenotype. Furthermore, apoptotic cells are capable of actively inhibiting the inflammatory program. For example, the inflammatory response induced by lipopolysaccharide (LPS), a component of the cell wall of Gram-negative bacteria, is strongly attenuated by preincubation of macrophages with apoptotic cells (46–48). First, after the exposure to apoptotic cells, an immediate-early inhibition of macrophage pro-inflammatory cytokine gene transcription can be detected (46, 47), subsequently, both nuclear receptors (38) are activated and, as it was first recognized, soluble mediators, such as transforming growth factor (TGF)-β and interleukin (IL)-10, are released (48). These mediators act *via* paracrine or autocrine mechanisms to strengthen and maintain the anti-inflammatory state (47, 48). The aim of this review is to highlight how these efferocytotic processes are coupled to the anti-inflammatory mechanisms provoked simultaneously by apoptotic cells, since efficient apoptotic cell removal and induction of immunologic tolerance by apoptotic cells are the two crucial mechanisms that prevent chronic inflammation and autoimmunity (49).

## Apoptosis is an Immunologically Silent Form of Cell Death

Increasing evidence indicates that the caspase-dependent apoptosis is unique in a sense that it is an immunologically silent form of cell death (50). Whereas necrotic cells typically provoke inflammation, apoptotic cells generally do not. What is more, even if apoptotic cells enter secondary necrosis and are leaking their cellular contents, they retain this anti-inflammatory state, in contrast to cells that have entered necrosis directly (51, 52). This suggests that significant alterations to cellular composition must occur during apoptosis to diminish the activity of danger-associated molecules (DAMPs), and even if these are accidentally released, their pro-inflammatory activity is destroyed. There are several examples recently discovered that underline this assumption.

Genomic DNA is considered to be an important DAMP capable of initiating dendritic cell (DC) maturation and the initiation of immune responses to coadministered antigens (53). Conversely, hydrolysis of DNA with endonucleases strongly attenuates its immune-activating properties (54). In apoptotic cells, genomic DNA undergoes extensive hydrolysis to small ~200 bp fragments due to the actions of a caspase-activated DNAse (55). If DNA cannot be degraded by either apoptotic or phagocytic endonucleases, autoimmunity develops (55), which is related to incompletely digested DNA persisting in macrophages leading to the activation of the RIG-I/IRF-3 pathway which senses cytoplasmic DNA fragments (56). In addition to the caspase-dependent degradation of genomic DNA, activated caspase-8 itself also interferes with the RIG-I/IRF-3 pathway by proteolytically inactivating RIP kinase 1, a key signaling component of the RIG-I complex, and thereby attenuating expression of IRF-3-inducible genes that include interferons and other inflammatory factors (57). Apoptotic caspases, *via* interfering with the cGAS/STING pathway, prevent the induction of type I interferons also by mitochondrial DNA, which can be released into the cytosol during apoptosis following permeabilization of the mitochondrial outer membrane (58).

High mobility group 1 (HMGB1) is another well-known DAMP. It functions in the cell nucleus as an architectural chromatin-binding factor *via* bending DNA and facilitating this way the buildup of protein complexes on specific DNA sequences. But it associates only loosely with the DNA, and can be passively released by necrotic or damaged cells (59). In apoptotic cells, however, HMGB1 remains bound firmly to chromatin due to the generalized hypoacetylation of histones, another caspase-dependent event (59). Furthermore, caspases also inactivate HMGB1 indirectly *via* cleaving the mitochondrial protein p75NDUF. This event triggers a burst of reactive oxygen leading to oxidation of a critical cysteine residue on HMGB1 that neutralizes its pro-inflammatory activity (60). And finally, IL-33, a recently discovered alarmin, is also cleaved by caspases loosing this way its DAMP activity (61).

## Apoptotic Cells Release Anti-Inflammatory Molecules

Apoptotic cells not only fail to be strongly immunogenic but were found to release various anti-inflammatory molecules as well. Thus, T cells express TGF-β, and release it during apoptosis (62). TGF-β is a potent anti-inflammatory cytokine (63). Its anti-inflammatory effects are reflected in the observation that loss of TGF-β1 in mice leads to wasting syndrome accompanied by a multifocal, mixed inflammatory cell response and tissue necrosis, resulting in organ failure and death (64, 65). Both the initiation and resolution of general inflammatory responses involve TGF-β1. Thus, TGF-β1 stimulates monocyte migration and growth factor production (66). But after initiation of an inflammatory response, it also exhibits potent anti-inflammatory effects, including inhibition of neutrophil and T-lymphocyte adhesion to endothelium (67), downregulation of macrophages (66, 68), and antagonism of tumor necrosis factor-α (TNF-α) function (68). TGF-β is crucial also in the initiation of differentiation of the regulatory T cells (69), which play a key role in preventing the development of autoimmunity (70). TGF-β plays such an essential role in the initiation of regulatory T cell differentiation that prior to the initiation of thymocyte apoptosis no Treg cells can be detected in the thymus (71).

Apoptotic cells can also release IL-10 (72). IL-10, together with TGF-β, induces an immune suppressive response by promoting regulatory T cell formation and by affecting their function (73).

Annexin I, which is released by apoptotic cells and promotes efferocytosis (32, 33), was also found to significantly attenuate IL-6 signaling and the release of TNF-α from endotoxin-challenged monocytes by activating annexin A1 receptors of the formyl peptide receptor family and the consequent JAK/STAT/SOCS signaling (74). And, finally, apoptotic cells were found to release lactoferrin that inhibits the migration of neutrophil granulocytes and eosinophils toward the chemotactic signals released by apoptotic cells (75, 76). As a result, apoptotic cells induce only migration of macrophages, but other classes of professional phagocytes are not recruited by the apoptotic cells (76).

Increasing evidence indicate that several chemotactic signals released from the apoptotic cells can also serve as anti-inflammatory molecules. Thus, TSP-1 functions as a major activator of TGF-β1 (77) which is secreted to the extracellular matrix in an inactive form by being in non-covalent association with the latency-associated peptide (78). First, TSP-1 releases TGF-β1 from its latent form *via* interacting with the N-terminal region of latency-associated peptide, then it binds the mature TGF-β1. This interaction induces such a conformational change in the structure of TGF-β1 (79), which allows its binding to its receptor. Furthermore, TSP-1 or its protease-cleaved derivative can also bind to immature DCs (iDCs) and induce their tolerogenic state (80). Fractalkine was also shown to act as an inhibitor of LPS-induced TNF-α production by microglia cells (81), while lysophosphatidylcholine was reported to interfere with the LPS-induced NO and pro-inflammatory cytokine production by macrophages (82).

Once released from the apoptotic cells *via* a caspase-regulated pannexin channel (83), ATP is fast degraded to AMP very often on the surface of apoptotic cells (84) and then to adenosine by the cell surface 5′ nucleotidase of engulfing macrophages (85, 86). Adenosine then triggers macrophage adenosine A_2A_ receptors (A2ARs) to suppress the NO-dependent formation of neutrophil migration factors, such as macrophage inflammatory protein-2, *via* activating the adenylate cyclase/protein kinase A pathway (86). Interestingly, both adenosine A_2A_ and A_3_ (A3R) receptors are expressed by macrophages, and while A2ARs inhibit, A3Rs promote the release of neutrophil migration factors by engulfing macrophages (87). However, while A2AR expression increases (86), A3R is downregulated during the course of efferocytosis (87) potentiating and maintaining this way the anti-inflammatory effects of adenosine. In addition, adenosine, by triggering adenosine A3R, contributes also to the chemotactic navigation of macrophages toward the apoptotic cells driven by apoptotic cell-derived chemotactic signals, thus facilitates the fast clearance of apoptotic cells (88). As a result, downregulation of A3R during clearance prolongs their presence around the apoptotic cells.

In an inflammatory milieu, adenosine strongly suppresses also the LPS-induced pro-inflammatory cytokine formation of monocytes and macrophages by activating A2ARs (89). In monocytes adenosine increases the expression of the Nr4A orphan nuclear receptor which then inhibits the transcriptional activity of nuclear factor-κB (NF-κB) known to play a determining role in initiating the transcription of numerous pro-inflammatory cytokines (90). The adenosine-triggered adenylate cyclase pathway in LPS-activated macrophages, on the other hand, upregulates the expression of dual-specific phosphatase 1 that interferes with the activation of LPS-activated MAP kinases (91).

## Apoptotic Cells Trigger Anti-Inflammatory Signaling Pathways by Activating Cell Surface Phagocytosis Receptors on the Phagocyte

Soon after it was discovered that PS recognition plays a central role in the uptake of apoptotic cells (92), it was also discovered that PS recognition in macrophages mediates also some of the anti-inflammatory responses observed during the uptake of apoptotic cells (93). This was proven by the observation that several anti-inflammatory responses provoked by apoptotic cells can be prevented by administration of annexin V, a naturally occurring PS binding protein (92), or are not induced by cells that do not express PS during apoptosis. In addition, in the induction of these anti-inflammatory effects, apoptotic cells can be replaced by PS liposomes (94). Some of the PS-induced anti-inflammatory responses are direct and can be detected as immediate inhibition of NF-κB transcriptional activity (46). Some others appear later, such as upregulation of the zinc finger nuclear factor, named GC binding protein (GC-BP) (95), or that of the Nr4a1 transcription factor (96). By binding to its promoter, GC-BP selectively inhibits IL-12 p35 gene transcription (95), while Nr4a1inhibits both NF-κB transcriptional activity and the induction of IL-12 (96). The induction of these transcription factors, however, might be macrophage type specific, because Nr4a1 induction is seen in peritoneal, but not in bone marrow-derived macrophages (96).

In addition to these intracellular anti-inflammatory effects, PS is also responsible for triggering TGF-β1 secretion from engulfing macrophages. Thus, apoptotic PLB-985 cells that are unable to express PS during apoptosis, fail to trigger TGF-β1 production, while PS directly transferred onto the PLB-985 cell surface membranes or PS liposomes can restore the secretion of TGF-β1 (94).

Since PS is recognized by a number of phagocytic receptors and opsonins that span a wide range of gene families, it is very likely that they induce immune suppression and tolerance *via* overlapping and non-overlapping mechanisms. Among the PS receptors, TIM-4 is not expected to transmit anti-inflammatory signals; since in the absence of a cytoplasmic tail, it alone cannot activate an intracellular signaling pathway (97). However, stabilin-2 was shown to be involved in inducing TGF-β1 release from engulfing macrophages (18), while MerTk was found to have a direct anti-inflammatory activity that suppresses NF-κB (98). The anti-inflammatory action of MerTk is independent of its effect on efferocytosis and is related to a signal transduction pathway that prevents the LPS-induced phosphorylation of IκB kinase and the consequent degradation of IκB (98). Whether other phagocytosis receptors also participate in the induction of the anti-inflammatory response of macrophages is still under investigation, but CD36 and α_v_β_3_ receptors do not seem to participate in it (99). Interestingly, however, loss of both MerTk and TG2 leads to pro-inflammatory cytokine production during efferocytosis (100, 101). But the pro-inflammatory cytokine production in this latter case might be related not only to an improper integrin signaling for which TG2 is a cofactor but also to the fact that TG2 is required for proper TGF-β activation by macrophages (102).

Phosphatidylserine receptors are expressed not only by macrophages but by other immune cells as well. As a result, apoptotic cells can transmit further immune silencing signals *via* activating PS receptors on those cells as well. For example, it has been shown that in the presence of apoptotic cells, iDCs do not induce expression of DC maturation-markers, such as MHC class-II, CD40, CD80, CD83, and CD86, even after challenge with CD40-signaling, monocyte-conditioned medium, LPS, or TNF-α (103–108). Furthermore, activation of PS receptors in human DCs by PS liposomes reduces their IL-12p70 secretion and the capacity to stimulate allogeneic T cell proliferation and to activate IFN-γ-producing CD4^+^ T cells (109). iDCs express MerTK, and activation of MerTK in iDCs triggers the phosphatidylinositol 3-kinase signaling pathway, which inhibits NF-κB activation and the consequent DC maturation (110). As a result, iDCs, which do not express MerTk or are treated with phosphatidylinositol 3-kinase inhibitors, do not respond to the immunosuppressing effect of apoptotic cells on LPS-induced pro-inflammatory cytokine formation (111). DCs also express Axl, another member of the TAM receptor family. The basal expression of Axl in DCs is very low, but it is significantly upregulated following TLR engagement. Axl, following activation, induces resolution of inflammation at the end of an inflammatory cycle (111). Ligation of the thrombospondin receptor CD36 also inhibits iDC maturation and function by suppressing the release of IL-12 and the secretion of high levels of IL-10 in response to DC-activation stimuli (105).

T cells, on the other hand, express the PS receptor TIM-3, another member of the TIM receptor family. In T cells, TIM-3, following PS exposure, transmits an immunosuppressive signal by sequestering lck, a critical tyrosine kinase participating in T cell receptor-mediated signal transduction (112).

In addition to PS, late apoptotic neutrophils were shown to express also pentraxin 3 (PTX3) in their membranes (113). PTX3 was shown not only to enhance their phagocytic removal during inflammation (113) but also to induce the expression of CD169 in macrophages (114), a molecule that interferes with the development of an autoimmune response (115).

## Nuclear Receptors Actively Suppress Pro-Inflammatory Cytokine Formation by Inhibiting NF-κB

Once apoptotic cells are taken up, they have to be fast metabolized by macrophages in order to ingest further apoptotic cells. Uptake of high amount of extracellular material might induce a metabolic stress in the engulfing macrophages, and engulfing macrophages respond to it in many ways by altering their metabolism. Interestingly, these metabolic adaptors also seem to contribute to the immune silencing processes as well. Thus, the amino acid metabolic-stress sensing protein kinase GCN2 was implicated to participate in the signaling pathways that lead to tolerance (116).

Similarly, the lipid sensing nuclear receptors that function during engulfment as transcriptional regulators of lipid metabolic processes also interfere with inflammatory processes, such as those initiated by TLR signaling in macrophages. As it was reviewed by Kidani et al. (38), under non-inflammatory conditions, NF-κB target genes are kept in an inhibited state by co-repressor complexes associated with their promoters. As a response to inflammatory signaling, proteins of these co-repressor complexes become ubiquitinated and subsequently degraded by the 19S proteasome. At the same time, NF-κB activated simultaneously by the inflammatory signals translocates into the nucleus, binds to promoters of inflammatory genes, and induces their expression. Ligation of PPARγ during efferocytosis prevents NF-κB-regulated gene expression by sustaining co-repressor binding on the promoters of NF-κB target genes. This is related to the fact that ligand binding to PPARγ induces conformational changes that allows for SUMOylation of its ligand-binding domain. SUMOylated PPARγ, subsequently, binds to the co-repressor complex and prevents its degradation by the 19S proteasome, thereby sustaining the suppressed state. LXR-mediated transrepression of NF-κB target genes operates *via* similar mechanisms involving SUMOylation of LXR by HDAC4, SUMO2, or SUMO3 as the E3-ligase (38).

Ligated PPARδ also interferes with the NF-κB transcription, but it operates through a different mechanism. BCL-6 is an evolutionarily conserved zinc finger transcription factor that acts as a sequence-specific repressor of transcription. Unliganded PPARδ sequestrates BCL-6 away from the promoters of inflammatory genes; while following ligand binding PPARδ releases BCL-6 leading to suppression of inflammatory gene transcription (117).

Ligated nuclear receptors are also anti-inflammatory because during efferocytosis they upregulate the expression of various phagocytic receptors, thus, enhance the clearance capacity of macrophages (118–122). The upregulation of some phagocytic genes by these receptors, such as MerTk by LXR, is direct, while that of others is mediated *via* retinoic acid receptor (RAR)-α (121, 122). In engulfing macrophages, activation of all lipid sensing receptors leads to the upregulation of retinaldehyde dehydrogenase expression, an enzyme responsible for retinoic acid synthesis (123). Retinoic acids, ligands for the RAR and RXR receptors, then can promote both the transcriptional activity of lipid sensing nuclear receptors and trigger that of the RARs (122). In addition, LXRs are also involved in the induction of the synthesis of polyunsaturated fatty acids, which are precursors for the production of pro-resolving lipid mediators (124). And finally, ligated nuclear receptors are also responsible for the upregulation of A2ARs that mediate the anti-inflammatory effect of adenosine (86).

Besides the lipid sensing nuclear receptors, the orphan nuclear receptor Nr4a1 has also been implicated to act as an anti-inflammatory molecule during efferocytosis (96). Nr4a1 seems to interfere with the TLR signaling pathway at two levels: (1) it can interact with TRAF6, a central adaptor molecule in the TLR signaling pathway. The interaction affects TRAF6 auto-ubiquitination leading to the suppression of NF-κB activation and to that of the subsequent transcription of pro-inflammatory cytokines (125). (2) It directly associates with the p65 subunit of NF-κB and prevents its binding to the κB promoter. However, this latter effect of Nr4a1 might be suspended by its LPS-induced p38α phosphorylation (126).

## Macrophages Respond to Apoptotic Cell Uptake by Releasing Anti-Inflammatory or Pro-Resolving Molecules

Since the first discovery that macrophages engulfing apoptotic cells release TGF-β and IL-10 (47, 48), numerous other anti-inflammatory molecules have been described to be released by engulfing macrophages. Many of these anti-inflammatory molecules are lipid mediators, such as PGE_2_, PGF_1α_, LXA_4_, or PAF, the synthesis of which is interestingly dependent on TGF-β (127). TGF-β at the same time inhibits the synthesis of pro-inflammatory lipid mediators. While in the case of TGF-β, it was found that activation of PS receptors can regulate its production (94), the regulation of IL-10 synthesis by macrophages engulfing apoptotic cells was not studied in detail. Annexin 1, however, is a known inducer of it in macrophages (128).

Retinoids produced during engulfment to enhance efferocytosis (122, 123) can also be released into the surrounding environment and contribute to the TGF-β-induced development of regulatory T cells. They act so by stabilizing TGF-β-induced Foxp3 expression under inflammatory conditions *via* inhibiting DNA methylation, which otherwise would lead to the silencing of the Foxp3 gene (129). Since regulatory T cells play a central role in preventing autoimmunity, release of TGF-β and retinoids by engulfing macrophages strongly contributes to the prevention of an autoimmune response build up following engulfment of apoptotic cells (70).

In addition to producing anti-inflammatory mediators, engulfing macrophages converted from M1 pro-inflammatory macrophages to M2 CD11b^low^ macrophages (130) during inflammation are also capable of producing pro-resolving lipid mediators, such as resolvins, protectins, and maresin (131–133). These molecules are synthetized from ω-3 fatty acids *via* the 12/15-lipoxygenase pathway. Pro-resolving mediators are stimulators of resolution of inflammation. Each facilitates cessation of neutrophil transmigration, microbial phagocytosis, and engulfment of apoptotic neutrophils. They also promote the formation of CD11b^low^ macrophages, which in addition to producing pro-resolving lipid mediators, also contribute to the termination of efferocytosis and emigration to lymphoid organs (130) required for the proper termination of the inflammatory program. The conversion to pro-resolving macrophages is also facilitated by the atypical chemokine receptor D6 expressed on the surface of apoptotic neutrophils (134) and by the apoptotic cell uptake itself (135).

And, finally, apoptotic cells also modulate the function of immature iDCs in a way that they force them to secrete molecules which inhibit T cell function. For example, apoptotic cells induce in iDCs the secretion of interferon-γ that upregulates *via* autocrine and paracrine mechanisms their indoleamine 2,3-dioxygenase activity leading to the degradation of tryptophan into metabolites that inhibit T cell function (136). Apoptotic cells also induce the release of large amounts of nitric oxide from iDCs that impairs the T cell response (137). At the same time uptake of apoptotic cells by iDCs triggers TGF-β production, which also strongly contributes to the immunosuppressive effects of apoptotic cells *in vivo* (138). Interestingly, plasmacytoid DCs, which themselves cannot engulf apoptotic cells, can also initiate an apoptotic cell-induced anti-inflammatory response. However, their response is dependent on engulfing macrophage-derived soluble factors, including TGF-β (139).

## Concluding Remarks

**Figure 1:**
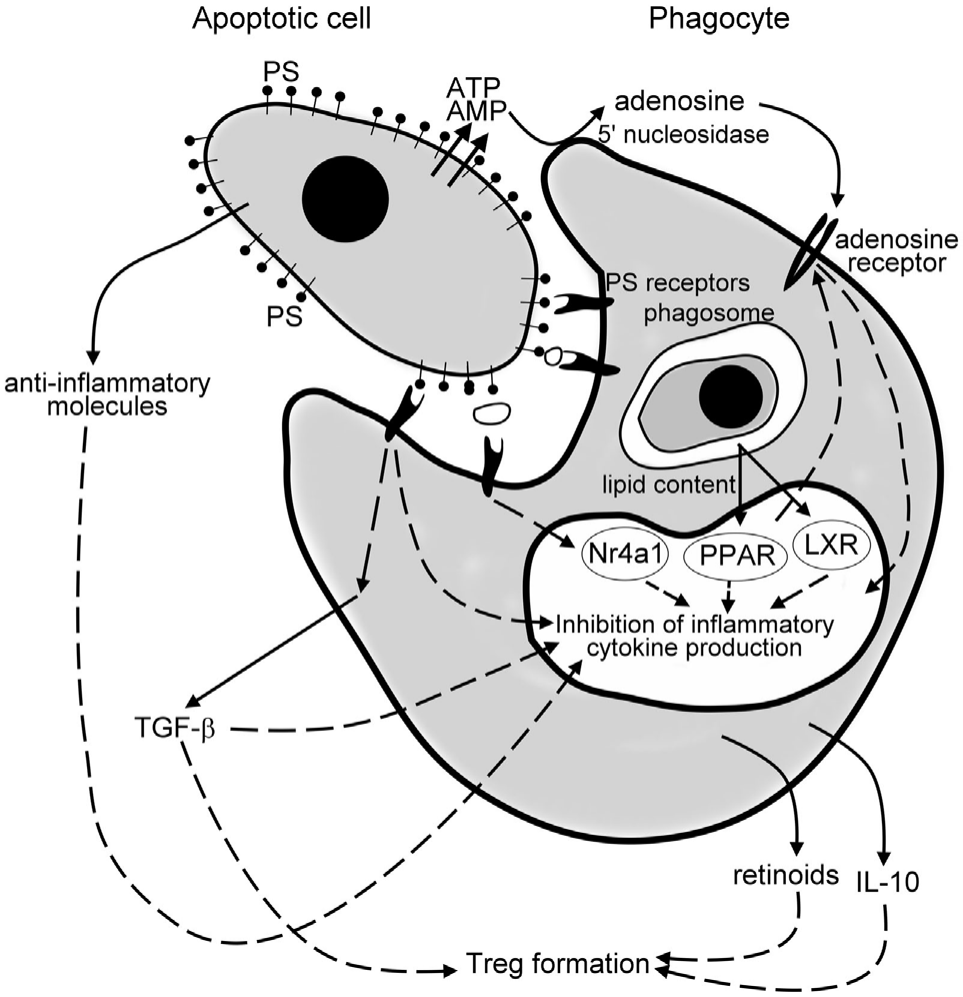
Mechanisms of the anti-inflammatory effects of apoptotic cells. Apoptotic cells release various anti-inflammatory molecules, such as transforming growth factor (TGF)-β, interleukin (IL)-10, annexin I, thrombospondin-1 (TSP-1), fractalkine to inhibit pro-inflammatory cytokine formation of phagocytes. Once released from the apoptotic cells *via* a caspase-regulated pannexin channel, ATP is fast degraded to adenosine by the cell surface 5′ nucleotidase of engulfing macrophages. Adenosine then triggers macrophage adenosine A_2A_ receptors to suppress the NO-dependent formation of neutrophil migration factors, or in an inflammatory milieu the lipopolysaccharide (LPS)-induced pro-inflammatory cytokine formation by phagocytes. Apoptotic cells express phosphatidylserine (PS), which activates various PS sensing phagocytic receptors. Many of these receptors initiate such intracellular signaling pathways that interfere with the pro-inflammatory cytokine formation of phagocytes. Following engulfment, the lipid content of the apoptotic cells activate the nuclear lipid sensing receptors [liver X receptors (LXRs) and perixosome proliferator-activated receptors (PPARs)], which in ligated form can also interfere with the NF-κB-driven pro-inflammatory cytokine formation. And, finally, engulfing macrophages release TGF-β, IL-10, and retinoids, which act in a paracrine or autocrine fashion to amplify and sustain the anti-inflammatory response and strongly contribute to the formation of the regulatory T cells to prevent the development of autoimmunity.

Apoptotic cell death is a determinant contributing factor to the cell turnover in most of the tissues. The silent removal of apoptotic cells maintains tissue integrity under healthy conditions and its anti-inflammatory nature contributes to the resolution of inflammation (Figure 1). Since the initial discovery that apoptotic cells possess potent immune suppressing effects on phagocytes of the immune system, numerous laboratories have initiated successful apoptotic cell-based therapies to induce donor-specific immunosuppression in transplantation (140). Though similar strategies were successful in mouse autoimmune experimental models as well, where apoptotic cells were given at the time of triggering of the disease (141), they would very likely fail in the treatment in chronic inflammatory diseases. This assumption is based on the observation that the establishment and/or progression of an increasing number of chronic inflammatory diseases seem to be coupled to improper phagocytosis of apoptotic cells (142). If the removal of apoptotic cells is delayed, their cell content is released and induces tissue damage leading to long-term inflammation or even to autoimmunity (143). During the past decade, we have understood that the development of chronic inflammation in these cases is related not only to the disturbed removal of dead cells but very likely also to the improper anti-inflammatory regulation that develops as a consequence of the improper clearance. Thus, enhancing efferocytosis and/or the related anti-inflammatory and pro-resolving mechanisms might provide a possibility in the treatment of those human chronic inflammatory diseases, in which impaired clearance of apoptotic cells appears as a driving or contributing force (49).

While the anti-inflammatory nature of apoptotic cell clearance is protective in healthy tissues, high PS expression of apoptotic tumor cells might promote tumor growth *via* suppressing immunity (93). Indeed, increasing evidence indicate that systemic administration of annexin A5 or other PS ligands that cover PS and block PS-mediated apoptotic tumor cell signaling may slow down tumor progression *via* interfering with the immunosuppressive properties of apoptotic tumor cells and tumor-derived microvesicles (144, 145). Thus, it was suggested that in combination with radio- or chemotherapy, annexin A5 could be used as a natural adjuvant to increase the immunogenicity of dying tumor cells thereby promoting the development of an anti-tumor immune response (93, 146).

And, finally, it is worth to note that though apoptotic cells are generally anti-inflammatory, cancer cells undergoing apoptosis in response to specific anticancer therapies can be immunogenic, if they emit precise DAMPs in a spatiotemporally defined fashion. Some of these DAMPs activate DCs during engulfment, which then can prime CD4^+^ T cells, CD8^+^ cytotoxic lymphocytes, and γδT lymphocytes against one or several tumor-associated antigens (147).

## Author Contributions

ZSzondy, EG, BK, KK, and ZSarang contributed to the experiments related to the topic and write the paper together.

## Conflict of Interest Statement

The authors declare that the research was conducted in the absence of any commercial or financial relationships that could be construed as a potential conflict of interest.
